# A case report of mass conversion disorder associated with a local spirit narrative in Dhankuta, Eastern Nepal

**DOI:** 10.1097/MS9.0000000000004628

**Published:** 2025-12-19

**Authors:** Pradeep Khatiwada, Rashmi Aryal, Prasanna Subedi, Madhur Basnet

**Affiliations:** a Department of Psychiatry, BP Koirala Institute of Health Sciences (BPKIHS), Dharan, Sunsari, Nepal; b Department of Internal Medicine, BP Koirala Institute of Health Sciences (BPKIHS), Dharan, Sunsari, Nepal

**Keywords:** adolescent girls, conversion disorder, mass psychogenic illness, psychoeducation, school-based intervention, sociocultural factors

## Abstract

**Introduction:**

Mass psychogenic illness (MPI), also known as mass conversion disorder, is a condition where groups of individuals develop physical symptoms without an identifiable organic cause, often influenced by underlying psychosocial and cultural factors. In rural and semi-urban areas, such episodes are often misunderstood and attributed to supernatural beliefs.

**Case Presentation:**

We report an outbreak of MPI in a secondary school in Dhankuta, Eastern Nepal, affecting 25/170 adolescent girls aged 13–19 over a span of 6 weeks. The index episode involved four girls simultaneously experiencing crying, collapsing, and abnormal movements during a mathematics class. Subsequent cases followed a similar pattern, often triggered by academic stress, strict discipline, and circulating rumors of a young girl’s rumored death near the school premises. A structured intervention – including psychoeducation, individual and group counseling, and changes in school policy – resulted in a marked decline in cases within 5 days. At the 3-month follow-up, only 6/25 continued to exhibit occasional symptoms, and no new cases were reported.

**Discussion:**

This case emphasizes the interplay between psychological vulnerability, academic pressure, and sociocultural beliefs in the development and propagation of MPI. The exclusive involvement of adolescent females aligns with global patterns in similar outbreaks. The effectiveness of non-pharmacological interventions, including psychoeducation and school-based reforms, supports previous literature and highlights the importance of community-specific mental health strategies.

**Conclusion:**

MPI remains a significant public health concern in low-resource settings, particularly among adolescent populations. Culturally sensitive psychosocial interventions, coupled with stakeholder education and supportive school environments, are essential for effective management and prevention.

## Introduction

Mass conversion or mass hysteria, now preferentially termed mass psychogenic illness (MPI)/mass sociogenic illness, is a social phenomenon, showcasing collective anxiety due to a perceived threat. It can culminate into a cascade of physical symptoms suggestive of organic disease but invariably without an identifiable cause^[1,2]^. The first detailed study of this phenomenon seems to trace back to Hecker in 1844, calling it “St. John’s Dance,” as mentioned in an analysis of mass sociogenic illness in 2002^[3]^.

**HIGHLIGHTS**

- Mass psychogenic illness in 25 schoolgirls in rural Nepal linked to stress.
- Symptoms mimicked possession, with no medical cause found.
- Counseling and school reforms led to full recovery in 3 months.
- Highlights the need for culturally sensitive mental healthcare.
- Community awareness helped resolve and prevent future cases.

The International Classification of Diseases, Tenth Revision (ICD-10), defines dissociative (conversion) disorders as conditions characterized by a partial or complete loss of the normal integration between memories of the past, awareness of identity, immediate sensations, and control of bodily movements, in the absence of an identifiable organic cause^[4]^. Its manifestation in more than one person at a time is called mass conversion disorder. MPI refers to the rapid spread of illness signs and symptoms affecting members of a cohesive group, originating from a nervous system disturbance involving excitation, loss, or alteration of function, whereby physical complaints that are exhibited unconsciously have no corresponding organic etiology^[3]^. In Nepal, these cases often present themselves in the form of “chhopne/chhopuwa,” both individually as well as in clusters, which translates to possession^[5,6]^.

This perplexing psychosocial phenomenon has left an indelible mark on communities worldwide. Its study has been a subject of interest to many health workers due to the absence of any physiological cause for the symptoms. Often, the surrounding sociocultural beliefs make these cases out to be shrouded in mystery and the occult. In Eastern Nepal, a recent occurrence of mass hysteria raised significant concern among healthcare professionals and the local population.

A focused narrative search of the literature was conducted to contextualize this case of MPI. PubMed and Google Scholar were searched using key terms including “mass psychogenic illness,” “mass hysteria,” “mass conversion disorder,” “school outbreak,” “adolescent,” and “Nepal.” Relevant articles were selected based on their applicability to clinical features, sociocultural factors, and intervention strategies, and reference lists were reviewed to identify additional pertinent studies.

The objective of this case report is to systematically describe and analyze a school-based outbreak of MPI in Eastern Nepal, with particular attention to its clinical presentation, psychosocial and sociocultural precipitants, and response to intervention. Specifically, this report aims to characterize the pattern of symptom expression and propagation among affected adolescents, examine the influence of academic stressors and locally embedded cultural narratives on the emergence and maintenance of symptoms, and evaluate the short- and long-term effectiveness of structured psychosocial and school-level interventions in controlling the outbreak. By addressing these dimensions, the report seeks to contribute to a clearer understanding of how individual vulnerability, sociocultural context, and institutional environments interact in shaping MPI in low-resource, school-based settings.

## Case presentation

### Location

The incident occurred in a secondary school located in a rural village of Dhankuta, a hilly district in Eastern Nepal. As of 2023, the school has 450 students, with 38% girls (170). The village has a population of nearly 3000 people, most of them belonging to middle to low socioeconomic status.

### The first episode

An episode was defined as an acute, observable event characterized by the sudden onset of crying or unresponsiveness accompanied by abnormal involuntary body movements, occurring in the school setting, lasting from 30 min to 5 h, with preserved vital signs, normal neurological and general physical examination, partial or complete post-episode amnesia, and spontaneous resolution without residual deficits. The first incident in school occurred in a group of four girls studying in grade 9. It took place during the second class of the day when one of the four girls started crying loudly, then progressed to fall onto the ground and showcased abnormal body movements. Three other girls started crying almost simultaneously, fell into the ground and mimicked similar body movements. The episode lasted for about 2–3 h. The girls claimed to have no recollection of the episode upon waking up. During examination, no significant physical findings could be elicited. The girls were in the same social group and shared similar demographics and sociocultural backgrounds. The affected girls displayed a remarkable degree of synchronization in the onset and resolution of symptoms, suggesting a psychosocial origin rather than a physiological one.

### The epidemic

Following this incident, other school children, all females aged 13–19, began to experience similar episodes, notably occurring during the second period in which maths was taught and lasting anywhere between half an hour to 5 h. A total of 25/170 girls were affected within a span of 1 and a half months, as mentioned in Table 1, corresponding to an attack rate of approximately 14.7% among female students. The index episode involved four girls who developed symptoms simultaneously, followed by three additional cases on the same day after exposure to affected peers. These 7/25 cases constituted the primary cluster. Subsequently, 18 additional cases occurred over the following 2 weeks, representing secondary cases associated with observation and social exposure. No new cases were observed after the implementation of psychoeducation and school-based interventions. In an attempt to curtail this, the headmaster and teachers resorted to faith healers and priests, the latter of which conducted a “pooja” and taught them the “mrityunjaya mantra,” a verse believed to offer protection from evil spirits. However, the episodes continued to occur to the guardians’ concern.

**Table 1:** Demographic characteristics, clinical features, and outcomes of affected students

Variable	Findings
Total number of affected students	25
Age range (years)	13–19 years
Mean age	16 years
Gender distribution	Female: 100%, male 0%
Most common presenting symptoms	Fainting/collapse, crying, hyperventilation, tremors, altered consciousness
School grade(s) involved	8 and 9
Class period most frequently affected	Mathematics
Cases resolved after intervention	76%
Persistent or recurrent symptoms	24%

### The stressor

According to the interviews, most of the episodes occurred during mathematics class or around it. Students mentioned being physically reprimanded for any mistakes made during the class. There was also a prominence of local legends circulating about the gruesome death of a young girl a few years back. The girl was rumored to have been beaten and hanged to death nearby, where the girls’ washroom was situated. The washroom where it allegedly transpired had been demolished and reconstructed. However, the event and its particular details were fresh in almost every individual in that school as well as the village. The tense environment of the classroom, coupled with the stories, likely precipitated the episodes. It is likely that the episodes themselves added to the stress and induced episodes in the other girls suffering from the same condition.

### Intervention

Psychoeducation, group and individual counselling of the affected and supportive counselling for parents and other relatives and other non-affected people are common treatment approaches, as seen used in similar cases studied prior^[6–9]^. Here too, it was the mainstay of the approach to these incidences, according to the Manual for Management of Mass Conversion Disorder for Nepal^[10]^.

All affected students underwent initial clinical assessment, including vital sign monitoring and focused neurological and general physical examination. No abnormalities were detected on examination, and there was no history suggestive of acute infection, head injury, toxin exposure, hypoglycemia, or seizure disorder. Given the simultaneity of symptom onset, rapid recovery without residual deficits, normal physical findings, and strong temporal association with psychosocial stressors, further laboratory testing or neuroimaging was not pursued. The diagnosis was made clinically in consultation with a psychiatrist, in accordance with established diagnostic approaches for MPI, where investigations are reserved for cases with atypical features or focal neurological deficits. Episodes were classified as MPI based on well-established clinical and epidemiological criteria described in prior literature and consistent with ICD-10 and Diagnostic and Statistical Manual of Mental Disorders (DSM)-5-TR frameworks for conversion (functional neurological symptom) disorder.

At first, the principal and all schoolteachers were interviewed with the aim of getting the overview of the situation, knowing the incidences serially as well as having a better idea of the stressor and trigger for the students.

Then, a seminar was held in the school, for students both affected and non-affected, the teachers and family members by way of presentation, videos and pamphlets, specially highlighting the psychological nature of this illness and paying special attention to reduce the stigma around this illness.

The girls were all counseled individually and screened for anxiety and depression using the Generalized Anxiety Disorder (GAD)-7^[11]^ and Patient Health Questionnaire (PHQ)-9^[12]^ scale, respectively. They were also taught breathing exercises as well as other stress-relieving exercises to reduce any anxiety symptoms. An overview of managing problems, including listing them, choosing one, defining it, brainstorming on how to approach it, deciding and choosing between them, formulating an action plan and reviewing it was taught.

The headmaster and teachers were educated in the concept of primary as well as secondary gain and advised to encourage an early return to work and to pay only an adequate amount of attention to patients’ symptoms. The principal was advised to arrange a separate space for the girls during episodes to isolate them.

With the headmaster and teachers’ cooperation, there was a scheduling change made in the school; grades 8 and 9 had morning classes, and smaller grades were called after 10 o’clock. Breaks were added after every two classes. The teachers were encouraged to adopt a more lenient approach to students, and corporal punishment was prohibited.

Psychoeducation and counseling were also offered to the cases’ families about managing the episodes when they occur at home, as well as assuring that it is not a life-threatening illness.

The teachers also provided psychoeducation in different health posts on a weekly basis thereafter in order to prevent and reduce similar incidences throughout the district.

### Results

Following the interventions over a period of 5 days, improvement was immediately noted. 3 new cases occurred in the following week, after which no new cases occurred. In the first monthly follow-up following the counselling and psychoeducation, 1–2 episodes in total were noted per day, all of which were old cases. In a 3-month follow-up, 1–2 episodes occurred per week with no new cases arising. Only 6/25 afflicted were actively suffering from the attacks. 100% of the affected were young females, of the same socioeconomic class and same educational background. On 6-month and 1-year follow-up, no new episodes of such illness were noted, as mentioned in Table 2. The follow-up was done with doctors and psychiatrists. The symptoms were predominantly motor and similar throughout. There was no verifiable organic cause to the symptoms and showed a dramatic response to psychoeducation and other interventions.

**Table 2:** Timeline of events, interventions, and outcomes

Time point	Event	Summary of findings/actions
Baseline context	School and community characteristics	Rural secondary school in Dhankuta district, Eastern Nepal; 450 students (38% girls); village population ≈ 3000; predominantly low–middle socioeconomic status
Day 0	Index episode	Four grade-9 girls developed sudden crying, collapse, and abnormal movements with synchronous onset and resolution. Episodes lasted 2–3 h. No recollection afterward. No abnormal physical findings
Days 1–45	Progression to cluster outbreak	Similar episodes in additional female students (ages 13–19), mainly during second (mathematics) period. Duration 30 min–5 h. Total affected: 25 over 1.5 months
Early response	Faith-based rituals by locals and school	Faith healers consulted; pooja performed; “Mrityunjaya mantra” taught. No improvement seen
Stressors identified	Academic and sociocultural triggers	Physical punishment during mathematics class; circulating narrative of past student’s death near girls’ washroom; increased anxiety as episodes spread
Intervention day 1–5	Assessment of school environment	Principal and teachers interviewed to establish chronology, stressors, and school dynamics
	Psychoeducation program	Seminar for students, teachers, families using presentations, videos, pamphlets explaining psychogenic basis and reducing stigma
	Clinical evaluation	Individual counseling; GAD-7 and PHQ-9 screening; breathing and relaxation training
	Problem-solving training	Structured steps: listing problems → prioritization → definition → brainstorming → action plan → review
	Staff training	Teachers educated on primary/secondary gain; encouraged early routine return; isolation space arranged
	Structural changes	Morning classes for grades 8–9; younger grades after 10 a.m.; breaks added every two periods; corporal punishment banned
	Family counseling	Parents reassured and instructed on home management
	Community outreach	Weekly psychoeducation provided at local health posts
Week 1 post-intervention	Early outcomes	Immediate improvement; 3 new cases in first week; no new cases afterward
1-Month follow-up	Ongoing reduction	1–2 episodes/day among previously affected students only.
3-Month follow-up	Sustained improvement	1–2 episodes/week; no new cases; 6 of 25 still intermittently symptomatic
6- and 12-Month follow-up	Long-term outcome	No new episodes; stable remission; symptoms remained nonorganic and responsive to psychoeducation

## Discussion

The DSM Fifth Edition does not explicitly use the term *mass psychogenic illness*; however, it includes conversion disorder within somatic symptom–related disorders, characterized by neurological symptoms incompatible with known medical conditions and linked to psychological factors. This framework is applicable to the present outbreak, where the psychological and sociocultural context played a central role in symptom development and propagation.

In the current case, circulating narratives surrounding the alleged murder of a young girl near the school premises appeared to act as a potent psychological trigger. A strong female predominance has been consistently reported in similar outbreaks: Sapkota *et al* reported all but one affected individual to be female^[6]^, while a study from West Bengal documented 9 out of 15 cases among females^[13]^. Likewise, outbreaks among schoolchildren in multiple African settings predominantly affected adolescent girls^[8]^, and studies on pseudoseizures have demonstrated higher susceptibility among females^[14]^. Concordant with prior reports, all affected individuals in this outbreak were adolescent girls experiencing high academic stress and heightened anxiety. The rapid spread of symptoms within a defined social group and the absence of identifiable organic causes further highlight the shared psychosocial mechanisms underlying MPI^[15]^. Similar findings have been described in Western Nepal, where academic stress acted as a precipitating factor for conversion disorder among school-aged girls^[7]^.

The role of sociocultural beliefs was also evident. Widespread discussion of the alleged death created an atmosphere of fear and heightened emotional arousal, consistent with findings by Chowdhary *et al*, who identified the death of a close individual as a common trigger^[13]^. Reliance on traditional healers and religious practices is another recurring theme in MPI outbreaks: in rural Nepal, episodes were interpreted as possession by “witches”^[6]^, while in African school outbreaks, religious rituals were employed^[8]^. Unlike reports from West Bengal, where sensory triggers such as smell or sound were implicated, no such triggers were identified in this outbreak^[13]^.

The relative scarcity of recent Western literature on mass sociogenic illness may support the observation that outbreaks are more commonly reported in lower socioeconomic settings. Media and community narratives play a critical role in shaping outcomes, as scientific explanations have been shown to mitigate distress, whereas supernatural interpretations may exacerbate symptoms and social disruption^[16]^. Psychoeducation was the primary intervention in similar outbreaks in Western Nepal and proved effective in reducing cases over time^[7]^, a pattern consistent with the present study, where symptom frequency declined substantially by 3 months, with residual symptoms persisting in only a subset of students.

Despite the effectiveness of psychosocial interventions, refractory cases were observed, underscoring the need for further research into the underlying mechanisms and pathophysiology of MPI. While MPI remains a diagnosis of exclusion, outbreak settings characterized by clustered onset, normal clinical examinations, fluctuating symptoms, and identifiable psychosocial precipitants do not routinely require extensive investigations, which may risk reinforcing symptom persistence through medicalization.

This case offers several unique contributions to the existing literature on MPI. Unlike previous reports, this outbreak demonstrated a strikingly consistent temporal association with a specific academic period – mathematics class – highlighting the role of subject-specific performance anxiety and strict disciplinary practices as direct triggers. The event was further shaped by a distinct interaction between local cultural narratives and physical space, as the legend of a girl’s alleged death near the school washroom created an emotionally charged environment that amplified vulnerability despite the site’s reconstruction. The cluster was also unusually large and rapid, with 25 adolescent girls affected within 6 weeks, underscoring how structured school settings can accelerate transmission. Another unique aspect of this report is its comprehensive, multi-level intervention strategy, which combined psychoeducation and counseling with concrete institutional reforms, including timetable adjustments, prohibition of corporal punishment, and the creation of isolation spaces – an approach seldom detailed in earlier studies. The inclusion of validated screening tools (GAD-7 and PHQ-9) further strengthens the psychological assessment, especially within a rural, resource-limited setting. Additionally, the exclusive involvement of females in a co-educational school provides noteworthy insight into gendered sociocultural vulnerability. Finally, the clearly documented sequence of ineffective religious interventions followed by rapid clinical improvement, along with long-term follow-up extending to 1 year, contributes valuable evidence regarding prognosis, cultural dynamics, and the effectiveness of structured psychosocial management in MPI.

## Conclusion

This case highlights the significant role of sociocultural factors in MPI, with a marked predominance among adolescent females. Gender-specific vulnerability in this setting is likely influenced by sociocultural expectations, reduced autonomy, and heightened academic and social pressures, making dissociative symptoms a culturally acceptable manifestation of distress. Academic stress – particularly mathematics-related anxiety and punitive teaching practices – appeared to act as a major precipitating factor by lowering coping thresholds and facilitating dissociative responses during periods of peak stress, thereby enabling rapid symptom dissemination within the school environment.

Cultural narratives surrounding a perceived traumatic event, along with reliance on traditional and religious healing practices, further illustrate the influence of shared belief systems in shaping symptom interpretation and perpetuation. The persistence of symptoms in a small subset of students suggests underlying individual psychological vulnerabilities, supporting the view of MPI as a spectrum rather than a uniform, self-limiting condition. These findings underscore the importance of culturally sensitive, multidisciplinary interventions, with psychoeducation playing a central role in reducing fear, symptom reinforcement, and stigma. Early recognition and school-based stress reduction strategies may be critical in preventing future outbreaks in similar community settings.

## Data Availability

Yes, the data analyzed during the current study are publicly available, available upon reasonable request.

## Research registration unique identifying number (UIN)

N/A, as this is a case report.
